# Vector Contact Rates on Eastern Bluebird Nestlings Do Not Indicate West Nile Virus Transmission in Henrico County, Virginia, USA

**DOI:** 10.3390/ijerph10126366

**Published:** 2013-11-27

**Authors:** Kevin A. Caillouët, Charles W. Robertson, David C. Wheeler, Nicholas Komar, Lesley P. Bulluck

**Affiliations:** 1 St. Tammany Parish Mosquito Abatement District, 62512 Airport Rd. Bldg. 23, Slidell, LA 70460, USA; 2 Department of Biology, Virginia Commonwealth University, 1000 West Cary St., Richmond, VA 23284, USA; E-Mails: robertsoncw@vcu.edu (C.W.R.); lpbulluck@vcu.edu (L.P.B.); 3 Department of Biostatistics, Virginia Commonwealth University, 830 East Main St., Richmond, VA 23219, USA; E-Mail: dcwheeler@vcu.edu; 4 Division of Vector Borne Infectious Diseases, Centers for Disease Control and Prevention, 3150 Rampart Rd., Fort Collins, CO 80521, USA; E-Mail: nkomar@cdc.gov

**Keywords:** host-seeking rate, nestling, nest mosquito trap, arbovirus, West Nile virus

## Abstract

Sensitive indicators of spatial and temporal variation in vector-host contact rates are critical to understanding the transmission and eventual prevention of arboviruses such as West Nile virus (WNV). Monitoring vector contact rates on particularly susceptible and perhaps more exposed avian nestlings may provide an advanced indication of local WNV amplification. To test this hypothesis we monitored WNV infection and vector contact rates among nestlings occupying nest boxes (primarily Eastern bluebirds; *Sialia sialis*, Turdidae) across Henrico County, Virginia, USA, from May to August 2012. Observed host-seeking rates were temporally variable and associated with absolute vector and host abundances. Despite substantial effort to monitor WNV among nestlings and mosquitoes, we did not detect the presence of WNV in these populations. Generally low vector-nestling host contact rates combined with the negative WNV infection data suggest that monitoring transmission parameters among nestling Eastern bluebirds in Henrico County, Virginia, USA may not be a sensitive indicator of WNV activity.

## 1. Introduction

Numerous regional studies have demonstrated the importance of a single or few avian species in the local amplification of West Nile virus (family *Flaviviridae*, genus *Flavivirus*, WNV). Dubbed a super spreader, the American robin (*Turdus migratorius*; AMRO) has been shown to be the primary host responsible for the majority of infected mosquitoes in independent studies in Washington, DC [1], Illinois [2,3], and Colorado [4]. Though none of these studies has implicated a significant role for the Eastern bluebird (*Sialia sialis*; EABL) in the amplification of WNV, its close taxonomic relation to the AMRO (both members of the family Turdidae) suggest that WNV infection in EABL may be similar. West Nile virus host competence has been shown to vary more between taxonomic families than within them [5]. Several studies have documented WNV infection in EABL [6,7,8,9,10]. One study in particular documented seroprevalence as high as 36% in EABL, indicating frequent contact with WNV-infected vectors [6]. Hatch-year birds, in particular nestlings, may be particularly important to avian arbovirus transmission due primarily to their susceptibility to infection as well as being confined to nests, lacking protective feather coverage of adults, and exhibiting weak defensive behavior [11,12,13].

Zoonotic pathogens, such as WNV, have been suggested to exist within transmission *networks* where relationships among multiple host and vector species structure transmission rather than traditional transmission *cycles* among single host and vector species [14]. The frequency of vector contacts within these host networks is a critical parameter in understanding the transmission ecology of WNV [15]. Despite the importance of vector-host contact rates on the transmission of WNV, they remain relatively understudied due primarily to the logistical costs of inferring contact rates via blood feeding studies or direct observation of vectors seeking hosts [16,17]. The use of vector-host contact rates to predict local WNV activity would aid the planning and implementation of WNV prevention and control activities.

We hypothesized that interaction between vectors and hosts occupying primary WNV network nodes (super spreaders) such as AMRO may be modeled by the vector-nestling interaction in a closely related species, EABL. Though EABL and AMRO exhibit differences in their nesting behavior, EABL usage of nest boxes makes this species a widely studied and convenient model. Accordingly, we investigated the relationship between WNV infection of bluebird nestlings and vector-host contact rates among these nestlings to determine whether monitoring EABL-vector contact rates might predict local WNV transmission intensity. In addition, we assessed whether temporal variation in observed host-seeking rates among mosquitoes was associated with nestling abundance, vector abundance, or the ratio of nestlings to vectors.

## 2. Experimental Section

### 2.1. Study Site

**Figure 1:**
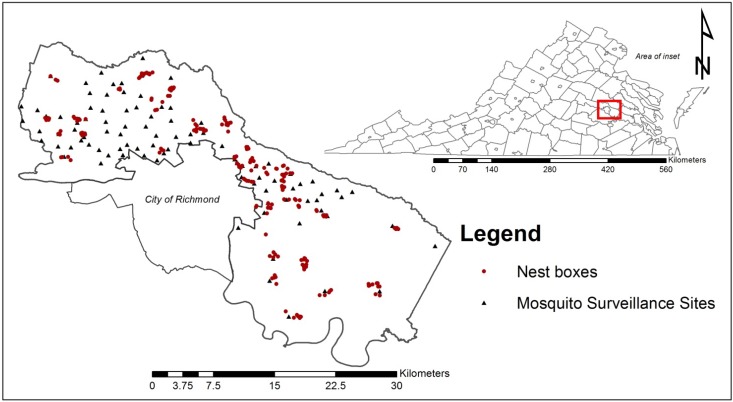
Study site map of Henrico County, Virginia, USA showing location of the 210 nest boxes (red dots) and 96 mosquito surveillance sites (black triangles) monitored during the 2012 mid to late avian nesting season.

The study took place throughout suburban and rural Henrico County, Virginia, USA (Figure 1). Bordering the city of Richmond, Henrico County is primarily a suburb of the city. Since 2001, the Henrico Standing Water Initiative (HSWI) has demonstrated annual evidence of WNV infection in birds, mosquitoes, and/or humans [18].

### 2.2. Nest Box Construction and Placement

We constructed and placed 210 nest boxes throughout the study area (Figure 1). Nest boxes were distributed in both historically active and inactive WNV transmission areas. We identified potential nest box sites by manually reviewing aerial imagery (updated December 2011) in ArcMap 10.0 (ESRI, Redlands, CA, USA) for the following criteria in order of priority: (1) within 1 km of HSWI mosquito surveillance sites, (2) having preferred EABL habitat (e.g., open tracts of lawn or meadow with occasional tree lines), (3) easily accessible by vehicle or foot, and (4) at least 150 m distant from another nest box. 

### 2.3. Nest Box Monitoring

We monitored nest boxes weekly from 21 May 2012 until the final nestling fledged on 16 August 2012. Occupancy status, species, presence and number of eggs, nestling condition and number, and the approximate age (days post-hatching) were recorded. Nest boxes containing nestlings were visited every 1–3 days to assess vector host-seeking rates and to monitor nestlings for WNV infection. To assess the temporal relationship among nestling abundance and vector host-seeking rates, we averaged nestling abundance across the study area for six two-week time intervals spanning the mid to late avian nesting season.

### 2.4. Nestling Swabbing and Virus Testing

We monitored nestling WNV infection status every four to five days while nestlings occupied the nest boxes. On 4th or 5th day post-hatching, EABL nestlings were banded with numbered bands (USGS permit #22751 and Virginia Department of Game and Inland Fisheries permit #041077) to identify each from their siblings. Animal handling protocols were approved by the Virginia Commonwealth University Animal Care and Use Committee (IACUC #AM10230). Nestlings of other species occupying the nest boxes (including House Sparrows (HOSP), House Wrens (HOWR), and Tree Swallows (TRSW)) were marked with unique paint markings on the tarsal claws. The oral-pharyngeal cavity of each nestling was swabbed with a Dacron-tipped swab every 4th or 5th day until they fledged, or were otherwise dispatched (predated, missing, or found dead). Specimens were transported on dry ice to the laboratory where the presence of infectious WNV was investigated by plaque assay using Vero cell monolayers overlaid with 0.5% agarose in 6-well plates [19].

### 2.5. Nest Mosquito Trapping

We monitored mosquito host-seeking rates using a modification of the original Nest Mosquito Trap (NMT) design [17] starting on 12 June 2013. This modification was made to increase the durability of the trap’s materials. Twenty-five modified NMTs were constructed of 0.59 cm-thick polycarbonate sheet plastic (Lexan^®^) instead of the original thin polypropylene container that had a tendency to warp with solar exposure.

**Figure 2:**
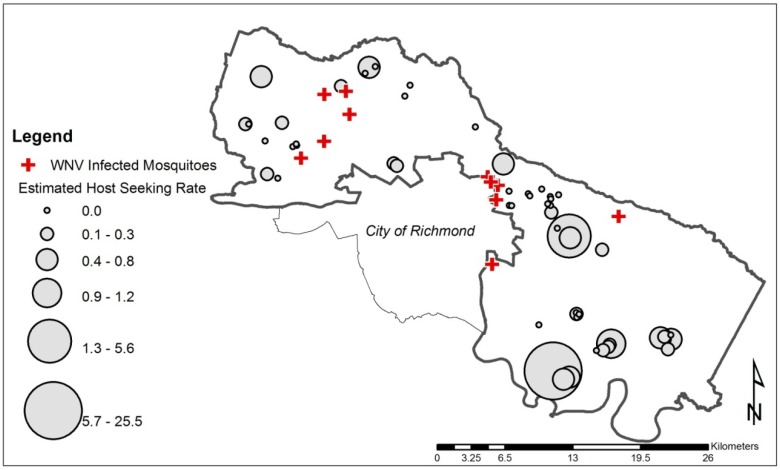
Nestling estimated host-seeking rates at 55 occupied nest boxes (graduated grey dots) and location of West Nile virus (WNV) positive mosquito pools (crosses) from 11 of 96 sites in Henrico County, Virginia, USA during the 2012 mid to late avian nesting season.

Only nest boxes that contained nestlings (55 of 210) were sampled with modified NMTs for host-seeking mosquitoes (Figure 2). Twice a week up to 25 NMTs/night were operated at nestling occupied nest boxes regardless of avian species. Traps were operated continuously overnight for 19–21 h. Nest Mosquito Traps were retrieved in the same order they were deployed to ensure equal running time between the subjects. In the laboratory, female mosquitoes were categorized using description of the blood meal taken, *i.e.*, unfed/empty, engorged, half engorged/half gravid, or gravid. Mosquitoes were then enumerated and identified to species using regional identification keys [20]. All collected mosquitoes were tested via RAMP^®^ (Response Biosciences, Vancouver, BC, Canada) for presence of WNV.

### 2.6. Calculation of Estimated Host-Seeking Rate

We estimated the per nestling host-seeking rate (hereafter estimated Host-Seeking Rate (eHSR) by adjusting the number of mosquitoes collected per nest box per trap night by a previously determined [17] laboratory capture efficiency ratio of (32.1%) and dividing the estimated scaled mosquito total by the number of nestlings within each nest box. Specifically, eHSR = (1/0.321) **×** observed mosquito count/number of nestlings.

### 2.7. Ambient Mosquito Abundance

We assessed ambient mosquito abundance to investigate its relationship with the NMT observed host seeking rate. Mosquitoes were collected from a network of 96 sites across Henrico County every two weeks as part of the county’s arbovirus surveillance program (Figure 1). The surveillance site locations had been previously chosen to optimize spatial coverage of surveillance data across Henrico County and to represent the spatial distribution of human density (*i.e.*, areas with high human populations have higher mosquito trap density). Carbon dioxide (1.3 kg of dry ice) and BG-Lure™ (Biogents) baited CDC light traps (Model 512, John W. Hock Company, Gainesville, FL, USA) and Frommer Updraft Gravid traps (Model 1719, John W. Hock Company) were used to monitor mosquito abundance and arbovirus infection. Collected mosquitoes were enumerated and identified to species using local morphological identification keys [20] by a trained technician. Mosquitoes were pooled into groups of 10–50 by species, collection site, and date. Pooled mosquitoes were tested for the presence of West Nile virus via RAMP^®^ (Response Biosystems). RAMP^®^ values exceeding 50 RAMP^®^ units were considered presumptive WNV positive and subsequently tested via RT-PCR for confirmation. The mosquito infection rate (number of infected mosquitoes/1,000, IR) was calculated using maximum likelihood estimates (PooledInfRate 4.0 [21]).

### 2.8. Statistical Analysis

We assessed the 55 site-averaged temporal associations among the mean eHSR and the 96 site-averaged WNV IR at six two-week intervals consistent with the temporal resolution of the IR data. These six biweekly rounds spanned the mid to late avian nesting season which has been associated with WNV amplification [15]. We also assessed whether the observed host-seeking rates were associated with mean ambient *Culex* spp. abundance, nestling abundance, and the ratio of ambient *Culex* spp. abundance to nestling abundance over the time periods using non-parametric Kendall’s tau correlations. Due to the sixth period (12–26 August) containing only two eHSR samples, we performed a secondary analysis excluding the final round of data. 

To evaluate the spatial relationship between eHSR and WNV status over the study period, we used a two-step modeling approach. Because nestling and mosquito monitoring sites were not spatially coincident, we first fitted a kriging model to predict the season-averaged eHSR at mosquito monitoring sites. eHSR was natural log transformed to follow a more normal distribution prior to kriging. We estimated a spherical variogram model and used an ordinary kriging model. The range of the estimated spherical variogram model was 15,103 feet. We then used the predicted transformed eHSR in a logistic regression model to explain the odds of WNV presence. Statistical analysis was conducted in the R computing environment [22].

## 3. Results and Discussion

Current WNV surveillance strategies including dead bird reporting, serologic monitoring of sentinel birds, and infected mosquito surveillance give little advanced indication of WNV risk to humans [23]. Limited warning of WNV activity across space and time significantly hinders disease intervention tactics. As enzootic WNV indicators, traditional surveillance strategies are sensitive, though not specific, precursors to human WNV infections [24]. Many statistical models incorporating climate, land use, and mosquito data have been developed to predict spatial distribution of WNV transmission risk [23,25,26], but few have been developed at local scales and fewer are used for operational targeting of control efforts. Due to these surveillance shortcomings, we investigated whether increases in vector host-seeking rates on nest box-occupying nestlings might precede enzootic transmission and therefore provide an earlier indication of local WNV transmission than existing surveillance methodologies.

A total of 127 mosquitoes were collected over 223 NMT sampling events in our study, of which 89.8% (117) were female. Due to the predominance of EABL occupying nest boxes, collection events were primarily performed on nest boxes containing EABL nestlings (94.3% of sample, 217 events). Other NMT collection events monitored vector-host contact rates on House sparrow (*Passer domesticus*; HOSP; 1.7%, four events), House wren (*Troglodytes aedon*; HOWR; 1.7%, four events), and Tree swallow (*Tachycineta bicolor*; TRES; 2.1%, five events). Ten species of mosquitoes (Diptera: Culicidae) were collected including: 38 (32.5%) *Culex erraticus* (Dyar and Knab), 53 (45.3%) *Cx. salinarius* (Coquillett), 10 (8.5%) *Cx. pipiens* (L.), one (0.9%) *Cx. pipiens/restuans* (L.), one (0.9%) *Cx. territans* (Walker), three (2.6%) *Cx.* species, four (3.4%) *Coquillettidia perturbans* (Walker), one (0.9%) *Aedes albopictus* (Skuse), four (3.4%) *Ae. vexans* (Meigen), one (0.9%) *Anopheles punctipennis* (Say), and one (0.9%) *Ae. hendersoni* (Cockerell). The physiological status of the collected mosquitoes is presented in Table 1. None of the 117 female mosquitoes collected from nest boxes were positive for WNV by RAMP^®^.

**Table 1:** Physiological status of the 117 female mosquitoes collected by the Nest Mosquito trap.

Species	Non-Engorged		Gravid		Blood Engorged		Half Gravid/Engorged	
	#	%	#	%	#	%	#	%
*Aedes albopictus*	1	100.0	0	0	0	0	0	0
*Aedes hendersoni*	1	100.0	0	0	0	0	0	0
*Aedes vexans*	4	100.0	0	0	0	0	0	0
*Anopheles punctipennis*	1	100.0	0	0	0	0	0	0
*Coquillettidia perturbans*	3	75.0	0	0	1	25.0	0	0
*Culex erraticus*	36	94.7	1	2.6	0	0	1	2.6
*Culex pipiens*	3	30.0	0	0	7	70.0	0	0
*Culex pipiens/restuans*	1	100.0	0	0	0	0	0	0
*Culex salinarius*	48	90.6	0	0	1	1.9	4	7.5
*Culex species*	3	100.0	0	0	0	0	0	0
*Culex territans*	1	100.0	0	0	0	0	0	0

A total of 697 oral-pharyngeal swabs were taken from 319 individual nestlings within 74 individual broods from the 55 occupied nest boxes. Proportional to the species occupation of nest boxes, most of the swabs were taken from EABL (90.6% of sample; *n* = 289 nestlings). Additional nestling species swabbed included HOSP (1.3%; *n* = 4), HOWR (1.9%; *n* = 6), and TRES (3.1%; *n* = 10). A mean of 2.1 swabs per nestling ± 0.06 SE (Range 1–5) were taken over the course of the nestling periods. Seven nestlings found dead were also tested for the presence of WNV. None of the 697 swabs or seven dead nestling brain samples tested positive for WNV by plaque assay.

**Figure 3:**
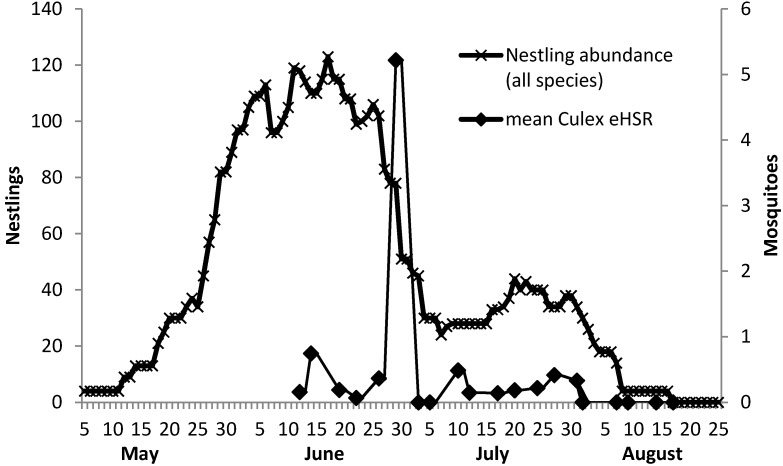
Temporal associations among *Culex* spp. estimated host-seeking rate and nestling abundance.

Estimated host-seeking rates calculated for *Culex* spp. mosquitoes collected in NMTs varied across nestling host species (Table 2), but the low number of sampling events for non-EABL birds did not allow for a statistical comparison of eHSR across host species. The *Culex* spp. eHSR mean/trap night across all time periods and spatial units was 0.7 ± 0.44 SE (*n* = 230). Host-seeking rates varied across the six biweekly time periods assessed (1 June–26 August) (Table 3 and Figure 3). Site aggregated mean eHSR by collection round peaked at 1.4 ± 1.13 (*n* = 55) during round 2 (16–30 June).

**Table 2:** *Culex* spp. nightly host-seeking rates across nestling species sampled.

Bird Species	*n* (Trap Nights)	Mean *Culex* spp. eHSR ^†^ ± SE
Eastern Bluebird	217	1.4 ± 0.34
House Sparrow	4	1.8 ± 0.63
House Wren	4	0 ± 0
Tree Swallow	5	9.3 ± 7.81

^†^ estimated host-seeking rate (eHSR) = (1/.321) **×** observed mosquito count/# nestlings; eHSR is a *per capita* (nestling) rate of mosquito landing that adjusts the number of collected mosquitoes by the laboratory efficiency rating of the collection device.

**Table 3:** Biweekly *Culex* spp. host-seeking rates (mean/trap night ± SE), *Culex* spp. abundance (mean/trap night ± SE), nestling abundance (mean/period ± SE), and West Nile virus mosquito infection rate (IR) in Henrico County, Virginia, USA, 2012.

Collection Round	eHSR Trap Nights ^1^	*Culex* spp. Ehsr ^1^	*Culex* spp. Abundance Trap Nights ^2^	*Culex* spp. Abundance ^2^	Nestlings	Culex: Nestlings	IR ^3^
1–15 June	43	0.4 ± 0.26	161	9.9 ± 1.09	107.2 ± 2.06	0.09	1.9
16–30 June	88	1.4 ± 1.13	172	10.2 ± 1.35	102.3 ± 3.75	0.1	0
1–13 July	34	0.1 ± 0.08	137	7.5 ± 1.21	33.9 ± 2.59	0.22	0
14–28 July	39	0.2 ± 0.08	187	9.7 ± 1.26	36.1 ± 1.27	0.27	1.7
29 July–11 August	24	0.1 ± 0.10	171	9.0 ± 1.95	19.4 ± 3.35	0.47	7.9
12–26 August	2	0 ± 0	206	5.9 ± 0.83	1.4 ± 0.53	4.15	2.1

^1^ Nest Mosquito Trapping began on 12 June 2013; ^2^ CO_2_ and BG-Lure™ (Biogents) baited CDC Light Trap and Frommer Gravid Traps; ^3^ Number of infected mosquitoes/1000.

**Figure A1:**
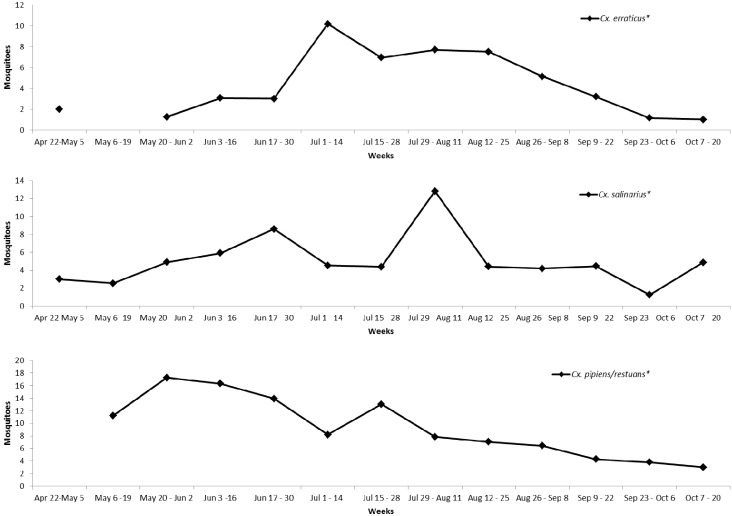
Seasonal periodicity of “ambient” mosquito abundance (2012) of selected Culex species mosquitoes in Henrico County, Virginia, USA.

Over the course of the study, the ambient abundance of *Culex* spp. mosquitoes collected by CO_2_-baited CDC light traps and Frommer Gravid traps at 96 sites peaked during the second collection round (16–30 June) at 10.2 mean/trap night ± 1.35 (SE) (*n* = 172) and was lowest at 5.9 ± 0.83 (*n* = 171) during the final period (12–26 August) (Table 3 and Figure 3). The seasonal abundance of *Cx. erraticus*, *Cx. pipiens/restuans* and *Cx. salinarius* is presented in the supplementary materials as Figure A1.

Sixteen of 435 (3.7%) pools tested via RAMP^®^ tested positive for WNV having RAMP^®^ values exceeding 50 units. These sixteen WNV-positive pools came from eleven sites within the 96 site surveillance network. Multiple WNV-positive mosquito pools were detected at two sites. The highest site-specific IR was 20.0 (1.23–112.83) (#infected mosquitoes/1000; 95% CI). Most (87.5%; 14 of 16) of the WNV-positive pools were comprised of *Cx. pipiens/restuans* mosquitoes (*n* = 256 pools). Single WNV-positive pools were detected also from *Ae. albopictus* (*n* = 171 pools) and *Cx. salinarius* (*n* = 5 pools). Across the season and county, *Cx. pipiens/restuans* had an infection rate of 3.1 (1.75–5.00). *Aedes albopictus* IR was 0.2 (0.01–0.77), while *Cx. salinarius* IR was 7.5 (0.49–38.43). Mosquito infection rate varied over time (0–7.93) (Table 2).

A comparison of the site-averaged data at six biweekly time periods for *Culex* spp. eHSR and covariates *Culex* spp. abundance, nestling abundance, *Culex* spp. abundance to nestling abundance ratio, and WNV IR revealed strong significant temporal associations. *Culex* spp. abundance (Kendall’s tau statistic 0.86; *p* = 0.016), and nestling abundance (0.86; *p* = 0.016) were strongly and significantly associated with eHSR, but Culex/Nestling (−0.73; *p* = 0.06) and WNV IR (0.41; *p* = 0.25) were not significantly associated. When round six was excluded from the analysis due to a paucity of eHSR samples, the strength of the observed temporal associations was mostly unaffected, although statistical significance decreased as expected. Excluding round six, *Culex* spp. abundance (Kendall’s tau statistic 0.80; *p* = 0.08) and nestling abundance (0.80; *p* = 0.08) were associated with eHSR, but not Culex/Nestling (−0.6; *p* = 0.23) or WNV IR (−0.32; *p* = 0.45).

The kriging-predicted, log-transformed eHSR was not associated with odds of WNV infection in mosquitoes. The estimated odds ratio of WNV infection among mosquitoes for eHSR was 0.51 (*p* = 0.73). The lack of an association is not surprising given the overall absence of copatterning in the WNV-positive sites and the larger observed eHSR values (Figure 2). 

Though it is well known that specific vertebrate hosts vary considerably in their competence to transmit WNV [27,28,29,30], insight into the effect of avian host life stage (*i.e.*, nestling, fledgling, juvenile, and adult) on WNV transmission is lacking. Specific host intrinsic factors such as pre-existing immunity, pathogen susceptibility, and infectiousness to vectors likely vary across host life stages [31]. Nestling birds have been suggested to be particularly important to avian arbovirus transmission due to being confined to nests, lacking protective feather coverage of adults, and exhibiting weak defensive behavior [11,12,13]. Hatch-year birds, excluding nestlings, were shown to be particularly important to WNV amplification in the Chicago, IL area in 2005–2006 [32,33]. Among five age cohorts of domestic goose (*Anser anser domesticus*), the youngest (six weeks old) were most affected (25% mortality rate) at a farm in southern Manitoba in 2002 [34].

Despite substantial effort to find viremic nestlings (swabbing nestlings every 4–5 days) and to monitor vector-host seeking rates (230 trap nights), we failed to detect the presence of WNV among vector or vertebrate populations occupying nest boxes. Low host-seeking rates and no WNV infection in mosquitoes seeking nestlings or in the nestlings themselves indicated an absence of WNV transmission among these bluebirds. Focal distributions of WNV-infected mosquitoes monitored by the local mosquito surveillance network at sites near to monitored nest boxes suggest that EABL nestlings play a limited or no role in the local WNV transmission network. Our findings are similar to a recent study that found that monitoring WNV infection in nestling HOSP did not provide advanced warning of WNV activity [35].

Shifts in host feeding from birds to humans associated with the end of the avian nesting season have been shown to increase simultaneous with human WNV risk [15]. Despite the annual consistency of this temporal association, few theories positing how WNV is amplified prior to this host feeding shift have emerged. Recently members of our research team demonstrated that a reduction in nestling hosts at the end of the nesting season with a coincident increase in vector abundance concentrated mosquitoes on few remaining nestlings [36]. The observed 36-fold increase in the vector-host contact rate due partially to host reduction may explain increased enzootic amplification of WNV from mid-July to mid-August. Similarly, Burkett-Cadena *et al.* [37] demonstrated that host feeding shifts across multiple taxonomic orders are likely the product of the timing of increased vector exposure during host reproductive periods. Our current analysis of the temporal patterns of host-seeking rates for mosquitoes supports our prior report [36] and Burkett-Cadena *et al.* [37] by providing evidence that temporal availability and absolute abundance of mosquitoes and nestlings mediate vector-host interactions. Despite this similar finding, we did not observe evidence of the concentration of mosquitoes on nestlings at the end of the nesting season as we did in 2010. Lack of evidence supporting the concentration of mosquitoes on the last remaining nestlings as observed in 2010 is further indirect evidence of the limited role of nestling EABL in WNV transmission in our study area. Habitat differences and species assemblages in the rural wetland environment sampled in 2010 likely account for the observed differences in host-seeking rates observed during this study in a suburban area.

Observed nestling host-seeking rates were temporally and spatially heterogeneous. On one trap night a nest box containing TRSWs experienced 11 mosquitoes per nestling while another nest box only 200 m distant with nestling EABL did not experience any mosquitoes. Similarly a nest box containing EABL nestlings that had no mosquitoes one night experienced an estimated 99 mosquitoes/nestling just two nights later. In this particular instance all of the nestlings except for one had died overnight. Spatial heterogeneity in vector-host contact rates has been documented in several other studies [1,3] and is assumed to play a primary role in the spatial distribution of WNV. Fine-scale temporal heterogeneity of host-feeding is less well studied, but is likely the result of fluctuating vector populations (e.g., pulsed emergence) and the distribution and availability of hosts [36].

Though we present substantial evidence suggesting that nestling EABL is not involved in WNV transmission in our study area, we cannot conclude definitively that nestlings of this species are not involved in the WNV network in Henrico County. The primary limitation of monitoring host-seeking rates using the NMT is the dependence on the geographic distribution of nest-box occupying avian species. Therefore, it is possible that our sample of the nest box-occupying avian population missed geographic WNV transmission foci. In addition, by sampling nestlings for virus shed orally every four to five days we may have missed infected birds. Interpreting statistical relationships among the temporal variables we present is also challenging given the low number of temporal sampling units in our study. Despite the failure to meet the nominal α = 0.05 threshold for statistical significance, the strength of the temporal relationships among eHSR and the variables of ambient mosquito abundance and nestling abundance suggest they independently structure host-seeking rates.

## 4. Conclusions

Despite high WNV activity in nearby areas, we did not detect WNV among nestlings or mosquitoes occupying next boxes in this study. Temporal variation in observed host-seeking rates was associated with vector and nestling host abundance. The lack of an observed role of nestling EABL in local WNV transmission in our study suggests that monitoring vector biting rates on nestling EABL is likely not a sensitive indicator of local human WNV risk.

## Figures and Tables

* Aggregated by week mean mosquitoes/trap night collected by BG-Lure™ (Biogents) and CO_2_-baited CDC light traps; † Aggregated by week mean mosquitoes/trap night collected by Frommer Updraft Gravid Traps.
